# Comparison of results of transcatheter femoral aortic valve replacement under local and general anesthesia: A protocol for systematic review and meta-analysis

**DOI:** 10.1097/MD.0000000000027085

**Published:** 2021-08-27

**Authors:** Xiangxiang Han, Shidong Liu, Jialu Wang, Hao Chen, Yang Chen, Bing Song

**Affiliations:** a The First Clinical Medical College of Lanzhou University; b Department of Cardiovascular Surgery, First Hospital of Lanzhou University, Lanzhou, China.

**Keywords:** General anesthesia, local anesthesia, meta-analysis, transcatheter aortic valve replacement

## Abstract

**Background:**

Traditionally, TAVR (Transcatheter Aortic Valve Replacement) has been performed under general anesthesia (GA). Thus GA facilitates the use of TEE (Transesophageal echocardiography), and the use of TEE is an important means to improve the quality of cardiac surgery and reduce postoperative complications. However, GA was also associated with prolonged mechanical ventilation, longer hospitalization and intensive care unit hours, and the need for positive inotropic agents. With increasing clinical experience and advances in transcatheter techniques, transfemoral TAVR may also be feasible under local anesthesia (LA). Studies have shown that LA can avoid hemodynamic fluctuations caused by general anesthesia and lung damage caused by positive pressure ventilation, and can also reduce medical costs.

**Methods:**

Two researchers independently read the titles and abstracts of the literature obtained. After excluding the studies that did not meet the inclusion criteria, they read through the full text of the remaining literatures to determine whether they truly met the inclusion criteria. When two researchers disagree on the included literature, the third researcher decides whether to include it or not. For literature with incomplete data, contact the author via email for unpublished data. The included studies were assessed by two researchers for the risk of bias, and cross-checked. Stata16.0 was used for meta-analysis. Heterogeneity was assessed by χ^2^ test and I^2^ quantification. Pooled analysis was performed by random effects model. Sensitivity analysis was performed by excluding references one by one. We will perform subgroup analysis based on data conditions.

**Results:**

In this study, high quality evidence was provided by selecting local anesthesia and general anesthesia during transfemoral transcatheter aortic valve replacement for patients with primary arterial stenosis.

**Conclusion:**

Local anesthesia provides anaesthetic-guided sedation that does not require intubation and is safe and effective. Local anesthesia may be a better alternative to TAVR under general anesthesia.

**Ethics and dissemination:**

The study does not require ethical approval.

**INPLASY registration number:**

INPLASY202170078

## 1 Introduction

Aortic stenosis (AS) is one of the most common acquired cardiac valvular diseases in the elderly and is usually the main cause of morbidity and mortality.^[1]^ After the onset of symptoms or cardiac function decline in AS patients, the mortality rate is high. If there is no intervention treatment, the 2-year mortality rate can reach 50% to 60%, but only conservative treatment is not effective.^[2]^ TAVR has been rapidly developed and popular worldwide due to its advantages of less trauma and quick recovery. Currently, more than 400,000 cases have been completed in more than 60 countries.^[3]^ TAVR was originally used as a minimally invasive aortic valve replacement procedure for high-risk or inoperable AS patients.^[4]^ Previous meta-studies comparing TAVR with surgical aortic valve replacement have confirmed that TAVR is a powerful tool for the treatment of severe AS at high or low moderate surgical risk, with much lower mortality compared to surgical aortic valve replacement.^[5]^ In fact, TAVR has also been widely used in severe AS patients with high risk of conventional surgery and anatomically suitable for TAVR.^[6]^

Traditionally, TAVR is performed under GA.^[7]^ GA can provide a controllable environment for the operator to use TEE easily. The use of TEE (Transesophageal echocardiography) is of great significance for timely intraoperative detection of myocardial diastolic function changes, valve annulus rupture, cardiac tamponema, determination of the location of implant prosthetic valve, determination of aortic regurgitation and perivalvular leakage, and is an important means to improve the quality of cardiac surgery and reduce postoperative complications.^[8,9]^ However, GA is also associated with longer periods of mechanical ventilation, longer hospitalizations and intensive care units, and the need for use of positive inotropic agents.^[10]^ More and more evidences indicate that with the increase of clinical experience and advances in transcatheter technology, transfemoral TAVR is also feasible under local anesthesia (LA).^[11]^ Previous studies have shown that LA can avoid hemodynamic fluctuations caused by general anesthesia and lung damage caused by positive pressure ventilation, and can also reduce medical costs.^[11]^ However, in some studies comparing anesthesia selection in patients with transfemoral TAVR, the criteria for choosing LA versus GA remain vague and often depend on institutional and surgeon preferences.^[12]^

Therefore, we conducted a systematic review and meta-analysis to compare the outcomes in AS patients with transfemoral TAVR at LA and GA.

## 2 Methods and analysis

### 2.1 Inclusion and exclusion criteria

#### 2.1.1 Type of study

Randomized controlled trials and cohort studies comparing the efficacy and safety of local and general anesthesia during transfemoral TAVR. The language is English only.

#### 2.1.2 Subjects

Patients with aortic stenosis requiring transfemoral TAVR.

#### 2.1.3 Interventions

When transfemoral TAVR was treated in the experimental group, the anesthesia mode was LA, while the control group was GA. There was no restriction on the type of valve (balloon dilatation or self-dilatation) and the specific mode of LA (selection of anesthesia drugs and anesthesia approach).

#### 2.1.4 Outcomes

The primary outcome measures were all-cause mortality (in-hospital, 30 days, and 1 year), postoperative stroke (in-hospital, 30 days), MI (in-hospital, 30 days), cardiac arrest, length of ICU care, and total length of hospital stay. Secondary indicators were surgery duration, duration of anesthesia, major bleeding event (including fatal bleeding event), vascular complications, new permanent pacemaker implantation, new onset of atrial fibrillation, and acute kidney injury.

#### 2.1.5 Exclusion criteria

1. case-control studies, case reports, meeting abstracts, comments and editorials, etc.;
2. The full text cannot be obtained, the data is incomplete, the data cannot be converted, there is no control group, and the calculation is wrong.

### 2.2 Search strategy

The PubMed, The Cochrane Library, Embase, and Web of Science databases were searched by computer, and the retrieval time was from the establishment to the end of September 2020. Key words: Transcatheter Aortic Valve Replacement, Transcatheter Aortic Valve implantation and General anesthesia. The strategy of combining subject words and free words was used to search. Literature traceability was carried out according to references included in the literature.

### 2.3 Data extraction and analysis

Two researchers independently read the titles and abstracts of the literature obtained. After excluding the studies that did not meet the inclusion criteria, they read through the full text of the remaining literatures to determine whether they truly met the inclusion criteria. When two researchers disagree on the included literature, the third researcher decides whether to include it or not. Data extraction includes: The first author, year of publication, study design, number of patients enrolled, baseline demographics, surgical data (duration of surgery, duration of fluoroscopy, duration of anesthesia), and hospitalization data (ICU) were collected. Duration of care (total length of hospital stay), postoperative complications (all-cause mortality, stroke, myocardial infarction, bleeding events, vascular complications, permanent pacemaker implantation), and quality evaluation information for included studies. For literature with incomplete data, contact the author via email for unpublished data.

### 2.4 Quality assessment

The risk of bias of the RCT was evaluated using the Cochrane Collabation Risk of Bias tool,^[13]^ and the Newcastle–Ottawa Scale (NOS) was used to evaluate the quality of the cohort study.^[14]^ The included studies were assessed by two researchers for the risk of bias, and cross-checked. If there was any disagreement, it would be discussed and resolved, and if necessary, it would be referred to the third researcher for decision.

### 2.5 Statistical analysis and assessment of heterogeneity

Stata16.0 was used for meta-analysis, and the count data were presented as relative risk and its 95% confidence interval. Mean Difference of 95% confidence interval was used as effect size for measurement data. Heterogeneity was assessed by χ^2^ test and I^2^ quantification. Pooled analysis was performed by random effects model. Sensitivity analysis was performed by excluding references one by one. Also *P* < .05 was considered statistically significant. We will conduct subgroup or sensitivity analysis to look for the potential causes.

### 2.6 Publication bias

Publication bias was evaluated by Stata 16.0 software funnel plot and Egger test. All the results were analyzed by funnel plot.

## 3 Discussions

With newer equipment and growing experience of operators, TAVR under LA is becoming a viable option for many hospitals. Switching from GA to LA is of great significance in terms of surgical risk, economy and other issues. The study confirmed that LA provides anaesthetic-induced sedation without intubation and is safe and effective. LA may be a better alternative to TAVR under GA, however, some large randomized controlled trials are needed to confirm this. In view of this, the selection of anesthesia method should also be combined with the requirements of anesthesiologists, surgeons and patients themselves, so as to provide the most favorable method for patients on the premise of ensuring the safe completion of the operation.

## Author contributions

**Investigation:** Jialu Wang.

**Methodology:** Xiangxiang Han.

**Software:** Hao Chen, Yang Chen.

**Supervision:** Bing Song.

**Validation:** Bing Song.

**Writing – original draft:** Xiangxiang Han, Shidong Liu.

**Writing – review & editing:** Xiangxiang Han, Shidong Liu.

## References

[1] Zhang G Luo J Huang X. Transcatheter aortic valve implantation: complications and anesthetic management. J Anes Periop Med 2018;5:215–20.

[2] Ramos M Quezada M Ayala R. Asymptomatic aortic stenosis in a geriatric population. The role of frailty and comorbidity in mortality. Rev Esp Cardiol (Engl Ed) 2021;74:167–74. 31882390 10.1016/j.rec.2019.11.006

[3] Smith CR Leon MB Mack MJ. Transcatheter versus surgical aortic-valve replacement in high-risk patients. N Engl J Med 2011;364:2187–98. 21639811 10.1056/NEJMoa1103510

[4] Cribier A Eltchaninoff H Bash A. Percutaneous transcatheter implantation of an aortic valve prosthesis for calcific aortic stenosis: first human case description. Circulation 2002;106:3006–8. 12473543 10.1161/01.cir.0000047200.36165.b8

[5] Sergi D Acconcia MC Muscoli S. Meta-analysis of the impact on early and late mortality of TAVI compared to surgical aortic valve replacement in high and low-intermediate surgical risk patients. Eur Rev Med Pharmacol Sci 2019;23:5402–12. 31298393 10.26355/eurrev_201906_18209

[6] Shah K Krinock M Thyagaturu H. Temporal trend, prevalence, predictors, and outcomes of pericardial diseases in patients undergoing transcatheter aortic valve repair. Cureus 2021;13:e16083. 34249583 10.7759/cureus.16083 PMC8248747

[7] Jabbar A Khurana A Mohammed A Das R Zaman A Edwards R. Local versus general anesthesia in transcatheter aortic valve replacement. Am J Cardiol 2016;118:1712–6. 27692595 10.1016/j.amjcard.2016.08.051

[8] Sheikh KH de Bruijn NP Rankin JS. The utility of transesophageal echocardiography and Doppler color flow imaging in patients undergoing cardiac valve surgery. J Am Coll Cardiol 1990;15:363–72. 2299078 10.1016/s0735-1097(10)80064-6

[9] Eltzschig HK Rosenberger P Löffler M. Impact of intraoperative transesophageal echocardiography on surgical decisions in 12,566 patients undergoing cardiac surgery. Ann Thorac Surg 2008;85:845–52. 18291154 10.1016/j.athoracsur.2007.11.015

[10] Motloch LJ Rottlaender D Reda S. Local versus general anesthesia for transfemoral aortic valve implantation. Clin Res Cardiol 2012;101:45–53. 21931964 10.1007/s00392-011-0362-8

[11] Fröhlich GM Lansky AJ Webb J. Local versus general anesthesia for transcatheter aortic valve implantation (TAVR)—systematic review and meta-analysis. BMC Med 2014;12:41. 24612945 10.1186/1741-7015-12-41 PMC4022332

[12] Maas EH Pieters BM Van de Velde M Rex S. General or local anesthesia for TAVI? A systematic review of the literature and meta-analysis. Curr Pharm Des 2016;22:1868–78. 26642777 10.2174/1381612822666151208121825

[13] Corbett MS Higgins JP Woolacott NF. Assessing baseline imbalance in randomised trials: implications for the Cochrane risk of bias tool. Res Synth Methods 2014;5:79–85. 26054027 10.1002/jrsm.1090

[14] Cota GF de Sousa MR Fereguetti TO Rabello A. Efficacy of anti-leishmania therapy in visceral leishmaniasis among HIV infected patients: a systematic review with indirect comparison. PLoS Negl Trop Dis 2013;7:e2195. 23658850 10.1371/journal.pntd.0002195 PMC3642227

